# Thickness Optimization of Highly Porous Flame-Aerosol Deposited WO_3_ Films for NO_2_ Sensing at ppb

**DOI:** 10.3390/nano10061170

**Published:** 2020-06-16

**Authors:** Sebastian Abegg, David Klein Cerrejon, Andreas T. Güntner, Sotiris E. Pratsinis

**Affiliations:** Particle Technology Laboratory, ETH Zurich, Sonneggstrasse 3, CH-8006 Zurich, Switzerland; sabegg@ethz.ch (S.A.); david.klein@pharma.ethz.ch (D.K.C.); andreas.guentner@ptl.mavt.ethz.ch (A.T.G.)

**Keywords:** environmental sensing, chemiresistor, flame aerosol technology, metal oxide, nanotechnology

## Abstract

Nitrogen dioxide (NO_2_) is a major air pollutant resulting in respiratory problems, from wheezing, coughing, to even asthma. Low-cost sensors based on WO_3_ nanoparticles are promising due to their distinct selectivity to detect NO_2_ at the ppb level. Here, we revealed that controlling the thickness of highly porous (97%) WO_3_ films between 0.5 and 12.3 μm altered the NO_2_ sensitivity by more than an order of magnitude. Therefore, films of WO_3_ nanoparticles (20 nm in diameter by N_2_ adsorption) with mixed γ- and ε-phase were deposited by single-step flame spray pyrolysis without affecting crystal size, phase composition, and film porosity. That way, sensitivity and selectivity effects were associated unambiguously to thickness, which was not possible yet with other sensor fabrication methods. At the optimum thickness (3.1 μm) and 125 °C, NO_2_ concentrations were detected down to 3 ppb at 50% relative humidity (RH), and outstanding NO_2_ selectivity to CO, methanol, ethanol, NH_3_ (all > 10^5^), H_2_, CH_4_, acetone (all > 10^4^), formaldehyde (>10^3^), and H_2_S (835) was achieved. Such thickness-optimized and porous WO_3_ films have strong potential for integration into low-power devices for distributed NO_2_ air quality monitoring.

## 1. Introduction

Every year, about 16 million Europeans are exposed to NO_2_ levels above those dictated by air quality guidelines [1]. This is alarming as NO_2_ (originating primarily from fuel combustion [2]) is one of the most harmful air pollutants [3], triggering several respiratory problems, such as wheezing, bronchitis, and even asthma already at ppb-level exposures [4]. To prevent adverse health effects, authorities have set strict exposure limits of about 100 ppb (1-h mean exposure) and 20 or 53 ppb (annual mean) in the EU [5] or the US [3], respectively. Considering frequently exceeded limits, distributed networks of detectors are urgently needed to recognize emission hotspots, as done already for other pollutants (e.g., trichlorofluoromethane [6]). If compact enough, such detectors could even serve as personal warning systems or support traffic control [7]. Nevertheless, reliable detection of ppb-level NO_2_ concentrations in the environment in the presence of hundreds of interferents (e.g., CO, CH_4_, H_2_S) and humidity is not trivial, requiring highly selective and sensitive but also inexpensive [7] detectors.

Sensors based on reduced graphene oxide [8], carbon nanotubes [9], and transition metal chalcogenides [10], among others, are promising for environmental monitoring due to their room-temperature operation. Nevertheless, they typically lack the required lower limit of detection [8,9,10] and selectivity [8,9] to reliably detect NO_2_ below 20 ppb in ambient air (Table 1). On the other hand, chemo-resistive semiconductive metal oxides (SMOx) are widely used in gas sensing [11] (e.g., WO_3_ [12], In_2_O_3_ [13], Co_3_O_4_ [14], ZnO [15], V_2_O_5_ [16]) and are attractive due to their reasonable stability [17], high miniaturization potential, and low production costs [18]. Despite their heating (e.g., 50–200 °C), low power consumption can be achieved (typically few tens of mW) when applied on μ-hotplates [18]. Such SMOx sensors typically consist of films assembled of nanoparticles that are deposited onto micro-electric circuitry [19]. The WO_3_ is a prominent material for NO_2_ detection due to its high sensitivity (e.g., down to 10 ppb [12]), optimum performance at low temperatures (typically below 150 °C, Table S1), excellent long-term stability (>1 year [20]), operation in humid conditions (e.g., 80% relative humidity (RH) [21]), and outstanding selectivity over environmental confounders (e.g., SO_2_, H_2_S, NH_3_, and ethanol) [22].

Film morphology optimization is an effective route to improve sensing performance [28]. Consequently, film thickness, porosity, pore size, and available surface area affect analyte’s penetration depth and interaction probability and, thus, sensor sensitivity. In fact, for sputtered WO_3_ films, the optimum NO_2_ response has been reported at ~85 nm thickness in the range of 40 to 200 nm [23]. Besides, the NO_2_ sensitivity of WO_3_ lamella stacks prepared by precipitation increase with porosity by adding hydrothermally-made SnO_2_ nanoparticles as spacers [29], though, chemical sensitization by SnO_2_ might also play a role. In addition, layer-by-layer inkjet printing has been used to obtain different thicknesses of Cu_2_O and CuO films for NO_2_ sensing; however, the effect of film thickness on sensor sensitivity and selectivity was not investigated [30]. Changes in film morphology (e.g., thickness, porosity), however, are accompanied typically by process-related changes, for instance, altered crystal size (e.g., during sputtering [23]) or additives [29]. As a result, also the transducer function in the sensing nanoparticles may be altered, making it difficult to associate increasing sensor performance unambiguously to film thickness. Cracks may also form during sensor film preparation (e.g., screen-printing [31] or doctor-blading [32]) that increases with increasing thickness [32] and can affect performance.

Flame spray pyrolysis (FSP) and deposition is an attractive method to generate highly porous films (e.g., 98% [33]) of nanoparticles with controlled film thickness. Nanoparticles are formed by a gas-to-particle conversion in the flame and can be deposited continuously by thermophoresis onto sensor substrates in a single step. Controlling thickness through deposition time is simple, while particle and crystal sizes do not depend on film thickness if particle formation is completed prior to deposition. Furthermore, flame-made sensing films are uniform, crack-free, and of high purity [33]. Such film formation can even be monitored by in situ resistance readout [34]. The FSP has been used already for the fabrication of Cr- [35] or Si-doped [36] WO_3_ sensors featuring outstanding acetone selectivity for breath analysis (e.g., fat burn monitoring during exercise [37] and dieting [38]) and in nearly-orthogonal sensor arrays [39] for human search and rescue [40]. Besides, wet-phase-deposited sensing films of flame-made Pt-doped SnO_2_ have shown stable sensor performance when tested for 20 days [41], while flame-deposited Pd-doped SnO_2_ sensors have featured stable performance for more than three months in a portable device for methanol and ethanol detection [42]. Furthermore, flame-made WO_x_ films (2.6 < x < 2.8) have responded to NO at 280–310 °C [43]. Such films should be accessed easily by NO_2_ as with CO [33] and provide a high surface area suitable to detect lowest pollutant concentrations, as demonstrated with doped SnO_2_ for other compounds (e.g., 3 ppb formaldehyde at 90% RH [44] or 5 ppb acetone at 50% RH [45]).

Here, we investigated the effect of porous WO_3_ film thickness for selectively sensing NO_2_ at realistic 50% RH. Nanoparticles were produced by FSP with crystal size and composition analyzed by X-ray diffraction and Raman spectroscopy. Highly porous films were obtained by direct deposition onto low-power μ-hotplate substrates. Film thickness was controlled through deposition time and characterized by cross-sectional focused ion beam scanning electron microscopy (FIB-SEM). The sensor performance at different thicknesses was evaluated at 125 °C [46] for sensing 3–100 ppb NO_2_ among common confounders—CO, H_2_, CH_4_, NH_3_, H_2_S, formaldehyde, acetone, methanol, and ethanol—at much higher concentrations and benchmarked to state-of-the-art solid-state NO_2_ detectors. These confounders were selected as they are among the critical ones outdoors [47].

## 2. Materials and Methods

### 2.1. Particle and Sensor Film Fabrication

A flame spray pyrolysis (FSP) reactor was used to prepare WO_3_ nanoparticles [35]. The precursor solution consisted of ammonium metatungstate hydrate (≥85% WO_3_ basis, Sigma Aldrich, Buchs, Switzerland) dissolved in an equivolumetric solution of ethanol (99.96%, VWR International, Dietikon, Switzerland) and diethylene glycol monobutyl ether (≥98.0%, Sigma Aldrich, Buchs, Switzerland) to obtain final tungsten molarity of 0.2 M [36]. The precursor was fed with 5 mL min^−1^, through the FSP nozzle, and dispersed by 5 L min^−1^ oxygen (99.5%, PanGas, Dagmersellen, Switzerland) at a pressure drop of 1.6 bar into a fine spray. Additionally, sheath oxygen was supplied at 5 L min^−1^ through an annulus surrounding the nozzle. A ring-shaped pilot flame of premixed methane (1.25 L min^−1^; 99.5%, PanGas, Dagmersellen, Switzerland) and oxygen (3.2 L min^−1^) ignited and sustained the spray-flame. The as-prepared nanoparticles were collected at 60 cm above the burner on a water-cooled glass-fiber (GF 6 257, Hahnemühle FineArt GmbH, Dassel, Germany) filtered by a vacuum pump and removed with a spatula. The sieved (250 μm mesh) powders were subsequently annealed at 500 °C for 5 h.

For sensor assembly, nanoparticles were flame-deposited for 1 to 18 min directly onto micromachined μ-hotplate sensor substrates (1.9 × 1.7 mm^2^) featuring a suspended membrane with an integrated heater and interdigitated electrodes (MSGS 5000i [48], Microsens SA, Lausanne, Switzerland) [49]. The substrates were mounted on a water-cooled holder and positioned at 20 cm above the burner. A shadow mask was applied to shield the contact pads. In situ annealing with a particle-free xylene flame (11 mL min^−1^) at 14.5 cm above the burner was applied to enhance adhesion and cohesion [50]. Subsequent annealing inside an oven at 500 °C for 5 h thermally stabilized the films further.

### 2.2. Powder and Film Characterization

Particle morphology was obtained by transmission electron microscopy (TEM, HT7700, Hitachi, Tokyo, Japan) at 100 kV. Crystal phases and sizes of the annealed WO_3_ powder were obtained by X-ray diffraction (XRD, AXS D8 Advance, Bruker, Billerica, MA, USA) operated at 40 kV and 30 mA at 2θ (Cu Kα) = 20 to 60°. The step size and scanning speed were 0.0026° and 0.62° min^−1^, respectively. Crystal phases were identified with reference structural parameters of monoclinic γ-WO_3_ (ICSD 80056), triclinic δ-WO_3_ (80053), and monoclinic ε-WO_3_ (84163), respectively, using the software Diffrac.eva V3.1 (Bruker, Billerica, MA, USA). Sample displacement was corrected by aligning the pattern to the peaks of crystalline NiO (~325 mesh, Sigma Aldrich, Buchs, Switzerland) that was added as an internal standard [51]. Corresponding crystal sizes and phase compositions were calculated by Rietveld refinement with the software Topas 4.2 (Bruker, Billerica, MA, USA) applied on the main WO_3_ peaks at 2θ = 20–36°. Additionally, Raman scattering was recorded with a 785 nm laser at 500 mW (operated at 1% intensity) in the range of 300 to 900 cm^−1^ using an exposure time of 1 min (InVia, Renishaw, Wotton-under-Edge, UK). The specific surface area (SSA) was measured by nitrogen adsorption (TriStar II Plus, Micromeritics, Unterschleißheim, Germany). The Brunauer–Emmett–Teller (BET) equivalent particle diameter was calculated with the density of WO_3_ (*ρ*_s_ = 7.16 g cm^−3^).

Film morphology was obtained by FIB-SEM (FEI Helios NanoLab 600i, Thermo Fisher Scientific, Hillsboro, OR, USA) operated at 2 kV. For cross-sectional images, a slice was cut into the sensing film by a focused beam of Ga^+^ ions at 30 kV. Rough milling to open the cross-section and subsequent polishing were performed at 2.5 and 0.77 nA, respectively. For the determination of film thickness, 40 measurements were taken across one sensing layer of the same sample using the application software of the SEM.

The porosity of flame-deposited WO_3_ films on Al_2_O_3_ substrates (20 × 20 mm^2^) was assessed by X-ray signal attenuation of the Al_2_O_3_ peaks by the WO_3_ film, as done with similar flame-deposited SnO_2_ films [33]. Therein, the X-rays penetrate the film with incident intensity $I_{in}$ and are attenuated by the film’s mass thickness s_s_ and bulk density *ρ*_s_, resulting in an emerging intensity $I_{em}$ following the exponential attenuation law [52]:

(1) $$\frac{I_{em}}{I_{in}}=exp\left(-\left[\frac{\mu }{\rho_{s}}\right]\rho_{s}\;s_{s}\right)$$

The $I_{in}$ was obtained from the signal of the empty substrate. The mass attenuation coefficient $\left[\mu /\rho_{s}\right]$ for WO_3_ at 8.04 keV (Cu Kα) was calculated as 13.59 m^2^/kg using the XCOM Photon Cross Sections Database [53]. Dense film thickness was calculated by correcting the mass thickness $s_{s}$ by the respective X-ray incident angle [33] and compared to the SEM film thickness to obtain the porosity. For the latter, the Al_2_O_3_ substrates were split using a wedge to obtain cross-sectional SEM images, and the thickness was evaluated at least 40 points across the cross-section. Finally, the average porosity was calculated from the main Al_2_O_3_ peak reflections at 2θ = 25.6, 35.1, 43.3, and 57.5°.

### 2.3. Gas Sensor Characterization

Up to four sensors were glued (PELCO Carbon Paste, Ted Pella, Redding, CA, USA) onto leadless chip carriers (Chelsea Technology, North Andover, MA, USA). Electrical connections were established by wire bonding (53xxBDA, F&S Bondtec, Braunau, Austria) before installing the chip in a stainless steel sensor chamber [44]. In specific, it consists of a cavity (18.1 × 16.6 × 18 mm^3^) arranged between a tubular gas inlet and outlet (Figure S1). Sensors were heated by the substrate’s integrated heater with a DC source (HMC 8043, Rohde & Schwarz, Munich, Germany). Sensing tests were carried out at 125 °C [46] and 50% RH (measured at 23 °C) with a total analyte gas mixture flow rate of 300 mL min^−1^. For that, dry and humidified synthetic air (C_n_H_m_ and NO_x_ ≤ 0.1 ppm, PanGas, Dagmersellen, Switzerland) were mixed by high-resolution mass flow controllers (EL-FLOW Select, Bronkhorst, Aesch, Switzerland), using a setup described in detail elsewhere [54], and measured just before the chamber inlet using a humidity sensor (SHT2x, Sensirion, Stäfa, Switzerland). That way, RH was maintained with small variation (e.g., 0.3% at 90% RH during 12 h [55]). Analytes were dosed into the dry synthetic airflow from dry calibrated gas bottles (all PanGas, Dagmersellen, Switzerland): NO_2_, H_2_S, CH_4_, NH_3_, acetone, methanol, ethanol (all 10 ppm in synthetic air); H_2_ (50 ppm in synthetic air); formaldehyde (10 ppm in N_2_); CO (500 ppm in synthetic air). Note that the O_2_ concentration in the gas mixture was reduced from 20 to 18 vol% for 1 ppm formaldehyde in N_2_; however, this should not affect the sensor performance [56]. All tubing was made out of inert Teflon and heated to 50 °C to avoid water condensation and minimize analyte adsorption.

The ohmic film resistances were continuously measured with a multimeter (Series 2700, Keithley, Cleveland, OH, USA) between the substrate’s interdigitated Pt electrodes. A picoammeter (Series 6487, Keithely, Cleveland, OH, USA) was used to read-out resistances > 100 MOhm. The analyte response for reducing gases was defined as S = R_air_/R_analyte_ − 1, where R_air_ and R_analyte_ are the film resistances in the absence and presence of analyte [54], respectively. For oxidizing gases, it was defined as S = R_analyte_/R_air_ − 1. Response and recovery times were defined as the times needed to reach and recover 90% of the resistance change, respectively. Sensor sensitivity was defined as the derivative of the response with respect to the analyte concentration, according to DIN 1319-1:1995-01 5.4.

## 3. Results and Discussion

### 3.1. WO_3_ Nanoparticle Characterization

**Figure 1:**
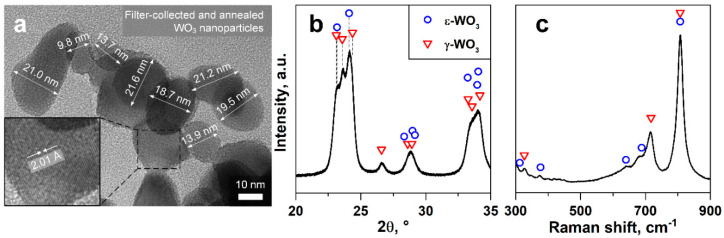
Material characterization. (**a**) TEM image with marked primary particle sizes in nm with inset at higher magnification, showing the lattice orientation and spacing in Å. (**b**) XRD pattern and (**c**) Raman spectrum of the filter-collected and annealed WO_3_ particles. Reference peak positions of monoclinic γ-WO_3_ (triangles, ICSD 80056) and ε-WO_3_ (circles, 84163).

The filter-collected and annealed powder consisted of agglomerated WO_3_ nanoparticles, as shown by TEM (Figure 1a). The primary particles were rather spherical and highly crystalline, as indicated by the well-developed lattice fringes (Figure 1a, inset), consistent with the literature [36]. These fringes expanded over entire particles, suggesting monocrystallinity. Particle diameters ranged between 10 and 22 nm, in agreement with the BET equivalent diameter of 20 nm, as calculated from the specific surface area (41.9 m^2^ g^−1^) obtained by nitrogen adsorption.

Figure 1b shows the XRD pattern in the relevant range of 2θ = 20–35° for WO_3_. Between 23 and 25°, three peaks characteristic for monoclinic γ-WO_3_ (triangles) were visible. Note that also triclinic δ-WO_3_ (Figure S2, squares) might be present, which featured almost identical peaks as the γ-phase. The γ-peak at 2θ = 24.37° was shifted to 24.14° (magnification in Figure S2). This suggested the presence of ε-WO_3_ (circles), featuring a peak at 2θ = 24.10°, in agreement with the literature [36]. Besides, the peak at 24.14° had higher intensity than that at 23.15°, characteristic for ε-WO_3_ (while they should be similar for γ-WO_3_). The peak at 23.60° might be affected as well due to the overlapping peaks—a characteristic for small crystal sizes. The ε-phase (typically only stable below −40 °C [57]) was obtained by FSP due to its high quenching rates capturing non-equilibrium phases, as shown also for BaCO_3_ [58], and was completely converted to γ-WO_3_ after annealing at 700 °C [59]. Neither crystalline impurities nor amorphous humps were identified by XRD (Figure S3). The latter indicated high crystallinity, as expected due to annealing.

Rietveld refinement estimated a phase weight ratio of γ to ε of about 1 to 1 with estimated crystal sizes (d_XRD_) of 23 and 16 nm, respectively, in agreement with TEM (Figure 1a) and BET. Note that the different phases could not be distinguished by TEM. A spacing of 2.011 Å (Figure 1 inset) was measured between the lattice fringes, which was similar to the (1 3 2) plane in γ-WO_3_ (2.017 Å) and the (−1 2 2) plane in ε-WO_3_ (1.994 Å). The crystal sizes compared rather well to reported d_XRD_ of 29 and 21 nm for γ- and ε-WO_3_, respectively, of flame-made WO_3_ in the literature [36]. Furthermore, crystal and particle sizes were smaller than the Debye length of WO_3_ (50 nm at 125 °C [60]), which should be favorable for gas sensing due to completely electron-depleted particles [61] and, especially, because the nanostructures were easily accessible due to the high film porosity.

The Raman spectrum supported the coexistence of γ- and ε-phases in the WO_3_ films (Figure 1c). The main reflection at 805 cm^−1^ could be assigned to both γ- [62,63] (triangles) and ε-phases [57] (circles), while peaks at 324 and 715 cm^−1^ were related only to the γ-phase [62,63]. The shoulders at 350–400 and 600–700 cm^−1^ suggested the presence of ε-phase peaks at 376, 642, and 688 cm^−1^ [57]. The fusion of the two peaks into a shoulder at 600–700 cm^−1^ rather than two distinct peaks (as observed for pure γ-phase) was most likely due to the small ε-phase crystal size (as estimated by XRD), which leads to peak broadening [64]. This shoulder increased with increasing ε-phase content [36].

### 3.2. Film Characterization

**Figure 2:**
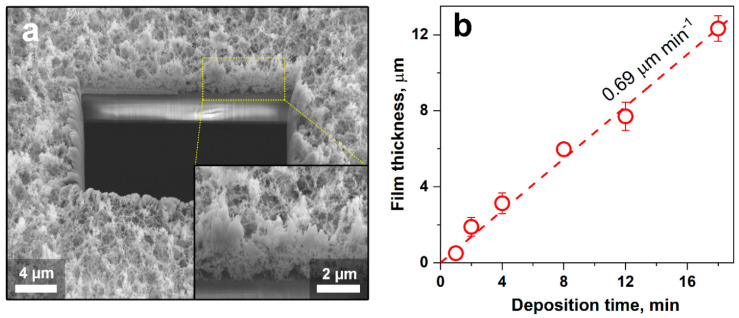
Film characterization. Cross-sectional SEM images of (**a**) porous 4-min flame-deposited film (on microsensor substrates) after cutting a square with a focused ion beam. Higher magnification is provided as inset. (**b**) The thickness of the deposited films as a function of deposition time. Symbols indicate average thicknesses, and error bars (some hidden behind the symbols) the standard deviations of, at least, 40 measured thicknesses per film. Dashed line represents a linear fit with indicated deposition rate.

Figure 2a shows a cross-sectional SEM image of a film after 4 min deposition. The film consisted of agglomerated WO_3_ nanoparticles, similar to the filter-collected ones (Figure 1a), forming a fine network with the large specific surface area—a characteristic for such flame-made films [33]. The resulting open film structure (Figure 2a, inset) provided large pores for rapid NO_2_ diffusion into the film. In fact, the porosity of the 4-min deposited WO_3_ film was 96.8 ± 0.1% (Table S2), in good agreement with similarly made Pt-doped SnO_2_ films [33]. Most importantly, the films were crack-free (e.g., compared to wet-phase deposited ones [32]), exhibited a homogeneous thickness across the entire cross-section, and their morphology was independent of deposition time (Figure S4). Please note that the more compact structures at the cutting edge were caused by melting during FIB.

**Figure 3:**
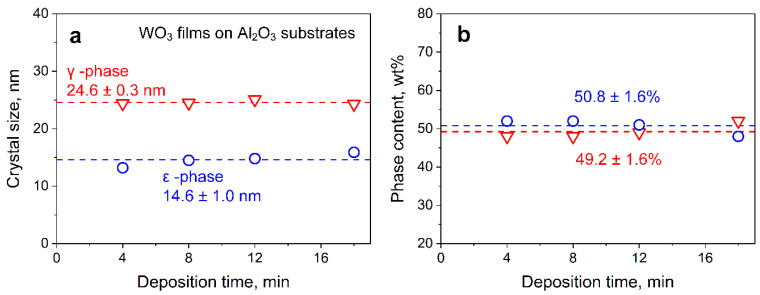
Effect of deposition time on WO_3_ composition. (**a**) Crystal sizes and (**b**) phase content for γ- (triangles) and ε-WO_3_ (circles) films on Al_2_O_3_ substrates (Figure S5) with comparable crystal size and phase composition to filter-collected WO_3_ powders. These films were prepared at identical deposition times to those on microsensor substrates that were too small and had too little mass for XRD. The films on Al_2_O_3_ were thicker and grew faster (Figure S6) than those on microsensor substrates due to the masking of the latter. Even for these on Al_2_O_3_, no reliable XRD evaluation could be obtained for 1 and 2 min deposition due to their weak WO_3_ signal.

The measured film thickness as a function of deposition time is shown in Figure 2b. Symbols and error bars represented average film thicknesses and standard deviations of, at least, 40 measured thicknesses across the whole cross-section for each deposition time, respectively. Error bars indicated the intra-sample variability. After 1 min of flame deposition, the film exhibited a thickness of about 0.5 μm. The thickness increased rather linearly with deposition time, up to 12.3 μm after 18 min, with a growth rate of 0.69 μm min^−1^. For thermophoretic deposition, such a linear trend could be obtained when the temperature difference between aerosol and film is constant [33], indicating efficient cooling. Besides, crystal sizes, phase contents (Figure 3), and porosity (Table S2) were not affected by deposition time, indicating that particle growth was completed before deposition [33]. Therefore, changes in sensing performance could be related quite reliably to film thickness.

### 3.3. Effects of Film Thickness on NO_2_ Sensing

**Figure 4:**
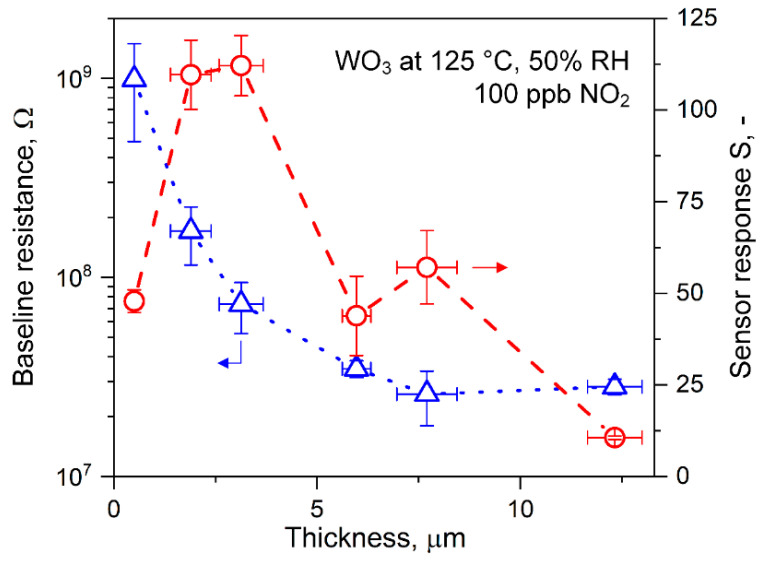
Effect of film thickness on sensor response. Baseline resistance (triangles) for flame-deposited WO_3_ films at 125 °C and 50% relative humidity (RH) (at 23 °C) and corresponding sensor responses to 100 ppb NO_2_ (circles). Symbols indicate the averages, and vertical error bars the corresponding variabilities of three identically produced sensors. Horizontal error bars represent the thickness variability of a sensing film (of 40 measurements).

Figure 4 shows the baseline resistance (triangles) for these flame-made WO_3_ sensors operated at 125 °C and realistic 50% RH. This sensing temperature was chosen, as identified previously for optimal NO_2_ sensitivity with hydrothermally prepared films [46]. The resulting power consumption was only 26 mW, making the sensor suitable for integration into battery-driven devices. The baseline resistance decreased steeply from about 10^9^ to 2.6∙10^7^ Ohm when the film thickness increased from 0.5 to 7.7 μm and leveled off thereafter. Such a reduction should be attributed to the additional conduction pathways (i.e., parallel resistances) with increasing film thickness, reducing the overall film resistance, in line with the literature [61]. The sensor baseline resistance drift was only 0.01% h^−1^ (Figure S7), which could be corrected during long-term operation with readily available algorithms (e.g., zero-calibration protocols [65]). The corresponding sensor responses (circles) when exposed to environmentally relevant 100 ppb NO_2_ [3] at 50% RH are shown in Figure 4. The NO_2_ was detected with an average response of 48 for the thinnest 0.5 μm films. Most remarkably, the response more than doubled to 110 and 112 when reaching an optimum thickness between 1.9 and 3.1 μm for 2- and 4-min deposited films, respectively. Error bars were typically <10%, showing good reproducibility of the process. Increasing responses with the thickness could be associated with the increasing number of reaction sites, intensifying chemiresistive interaction with NO_2_ [66]. For thicker films, however, the NO_2_ response decreased steeply to 44 at 6 μm thickness and even to 11 at 12.3 μm. Similarly, a decrease in ethanol sensor response with increasing film thickness was observed for flame-deposited SnO_2_ films [67] and doctor-bladed ones consisting of flame-made ZnO particles [32]. The response difference for the 6 and 7.7 μm thick films was within the error bars and not statistically significant. This response reduction could be attributed to compromised NO_2_ transport into the film due to increased diffusion resistance with increasing thickness [68]. Furthermore, the NO_2_ interaction in the upper film layers had less impact on the total resistance change than if the same reaction would occur in the lower layers, where also electrode effects might play a role [69].

The observed optimum (1.9–3.1 μm, Figure 4) for these flame-made and highly porous films occurred at a significantly larger thickness than for sputtered WO_3_ films (i.e., 85 nm) [23]. For the latter, however, crystal size depended on film thickness due to nucleation and growth during deposition [70] that influenced sensitivity [71]. Optima below 100 nm were also observed for sensing of other gases with compact films, e.g., ion-beam sputtered SnO_2_ (~7 nm for H_2_) [72] or atomic layer deposited SnO_2_ (~10 nm [73] or 2.6 nm [74] for CO). For such compact films, optimum sensitivity was expected if film thickness was in the range of the material’s Debye length (thus fully depleted layers), as shown for SnO_2_ [74]. This was in contrast to porous films, where the whole film was accessible for the analyte gas, and the transducer function in relation to Debye length was modulated by the particle/crystal size [75] rather than the film thickness. Nevertheless, for porous films obtained from wet methods (e.g., screen-printing), the crack formation was typically observed due to solvent evaporation [76] that could affect sensor results.

### 3.4. Low-ppb NO_2_ Sensing

**Figure 5:**
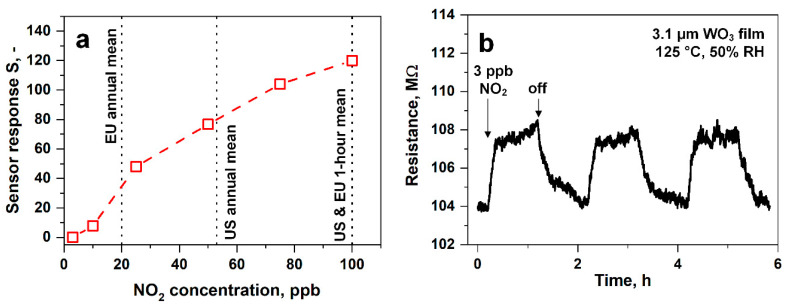
Low ppb NO_2_ detection. (**a**) Sensor responses of the thickness-optimized WO_3_ film (3.1 µm) in the relevant concentration range from 3 to 100 ppb NO_2_ measured at 50% RH (at 23 °C) and 125 °C. Threshold exposure limits from the EU [5] and the US [3] are indicated by dashed lines. (**b**) Film resistance upon consecutive exposure to 3 ppb NO_2_.

The legal NO_2_ limits are below 100 ppb [3], defining the required detection range for suitable sensors. Figure 5a shows the responses of the above thickness-optimized sensor (i.e., 3.1 μm thick) to 3–100 ppb NO_2_ at 50% RH. The sensor response increased continuously with a sensitivity of about 2 ppb^−1^ up to 25 ppb and thereafter with 1 ppb^−1^, suggesting the onset of saturation. This was orders of magnitude higher than graphene-based sensors operated at room temperature (~0.1 ppb^−1^) [77] or WO_3_ nanowires at 250 °C (~0.002 ppb^−1^) [78]. Such behavior was expected due to the nonlinear diffusion-reaction theory of such semiconducting metal oxide sensors [79]. Most importantly, hazardous concentrations exceeding the exposure guidelines in the EU and US (vertical dashed lines) could be clearly identified.

Figure 5b shows the change of film resistance when consecutively exposed to low NO_2_ concentrations of 3 ppb in 50% RH. The film resistance increased from about 104 to 108 MOhm when introducing 3 ppb NO_2_, as expected for oxidizing gases like NO_2_ that fill oxygen vacancies located on the surface of WO_3_ [80], leading to increasing film resistance. Thereafter, the resistance was fully recovered to its baseline. This resulted in a response of 0.04 with a high signal-to-noise ratio of 17. Besides, the same response was obtained upon consecutive exposure to 3 ppb, indicating good reproducibility. While sub-ppb NO_2_ has already been measured only in dry air [81], the present sensor detected 3 ppb at realistic humidity. This was considerably lower than other metal oxide-based sensors tested in relevant conditions (10–80% RH, Table 1) and commercial electrochemical sensors (lower limit of detection of 10 ppb [82]). Important to note, however, that the NO_2_ sensitivity of WO_3_-based sensors decreased with increasing RH, both at low (e.g., 25 °C [83]) and high temperatures (e.g., 300 °C [80]). Therein, H_2_O competed with NO_2_ for oxygen vacancies, as observed by in situ diffuse reflectance infrared Fourier transform spectroscopy and film resistance readout [80]. In fact, the NO_2_ response of WO_3_ sensors operating at 100 °C and dry air was halved by increasing the RH to 25%, however, influenced much less at higher RH (~30% for every 25% RH increase) [84]. This could be corrected by co-located RH sensors, as shown previously for sensing acetone, ammonia, and isoprene at 30–90% RH [40]. The detection of such low NO_2_ concentrations with flame-deposited WO_3_ films was associated with their highly porous and fine film morphology (Figure 2a and Figure S4). In fact, sensor responses at 150 °C increased in sputtered WO_3_ films after increasing porosity by annealing [85], and highly porous CuBr films showed an order of magnitude higher NH_3_ responses than denser ones [55]. The corresponding response and recovery times of 7.8 and 43.8 min, respectively, could be further decreased by transient response analysis [86].

### 3.5. NO_2_ Selectivity in Realistic Conditions

**Figure 6:**
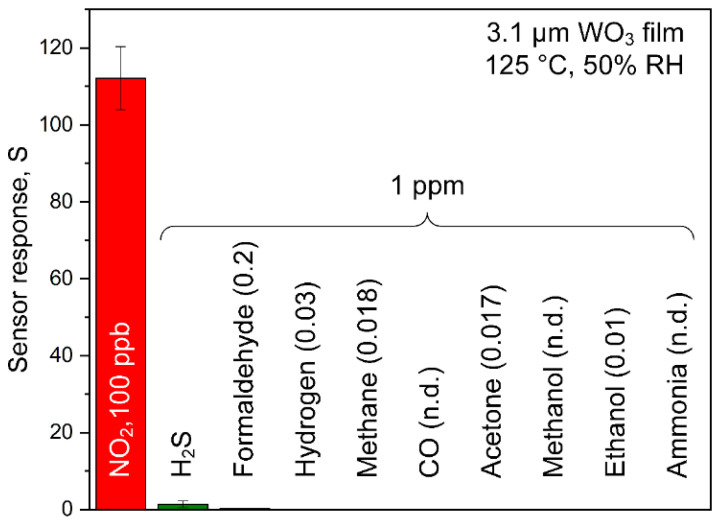
Selectivity. Sensor responses of the thickness-optimized 3.1 µm WO_3_ film to 100 ppb of NO_2_ and 1 ppm H_2_S, formaldehyde, hydrogen, methanol, CO, acetone, methanol, ethanol, and ammonia at 50% RH (at 23 °C). Please note that no response was detectable for CO, methanol, and ammonia.

In environmental monitoring, a key challenge is the selective detection of NO_2_ in the presence of interfering gases. Therefore, the responses of the above sensor (3.1 μm thickness) were tested for 100 ppb NO_2_ and its major confounders at much higher concentration (1000 ppb): H_2_S, formaldehyde, H_2_, CH_4_, CO (up to 40 ppm, Figure S8), acetone, methanol, ethanol, and NH_3_ at 125 °C and 50% RH (Figure 6). Excellent NO_2_ selectivities were obtained to CO, methanol, ethanol, NH_3_ (all > 10^5^), H_2_, CH_4_, acetone (all > 10^4^), and formaldehyde (>10^3^), indicating negligible cross-interference. Even to H_2_S, the selectivity was still >800. Please note that WO_3_ also showed high NO_2_ selectivity to other confounders, e.g., 150 for NO at 100 °C and 1 ppm [84]. Interestingly, also, the selectivity for most interferents was rather independent of film thickness, while for H_2_/ethanol and H_2_S, decreasing and increasing trends, respectively, were observed with the thickness (Table S3). These selectivities are most attractive for environmental NO_2_ monitoring, even if CO concentrations are present up to 40 ppm in rare situations, such as during traffic [87]. In specific, in the presence of 20 and 40 ppm of CO, the response was 0.02 and 0.07 (Figure S8), respectively, resulting in selectivities > 10^5^ (calculated at the same concentration with NO_2_). The selectivity could be further increased with filters preceding the sensor, e.g., size-selective membranes [88] or adsorption packed beds (e.g., Al_2_O_3_ to retain hydrophilic [89] or Tenax for hydrophobic compounds [49]).

Table 1 shows a selectivity comparison to state-of-the-art NO_2_ sensors at 10–80% RH and relevant sub-ppm NO_2_ concentrations. The present flame-made porous WO_3_ sensor showed superior selectivities over those prepared by sputtering (for NH_3_ and CO), drop coating (for acetone), and other coating methods (for ethanol), which could be attributed to their more compact film morphology and/or to the presence of ε-phase WO_3_ in the flame-made films (Figure 1b,c). Furthermore, Fe-doping [12] seems to decrease the selectivity significantly compared to pure WO_3_ sensors. Only brush-coated In_2_O_3_ [24] and SnO_2_/ZnO (UV excited) sensors [26] feature similar selectivity for most analytes. Finally, carbon-based (rGO, CNT) composites with metal-oxides [8,9,27] operated at room temperature are outperformed, featuring orders of magnitude lower selectivities (e.g., 9 vs. >10^5^ for CO).

## 4. Conclusions

Highly porous, crack-free, and homogeneous WO_3_ sensing films with controlled thickness were created by FSP. The films consisted of mixed-phase γ- and ε-WO_3_, as revealed by XRD and Raman spectroscopy. Their thickness was controlled through the deposition time without affecting the crystal size or phase content of WO_3_. Such flame-deposited films at an optimal thickness (i.e., 3.1 μm) exhibited an order of magnitude higher NO_2_ response than the thickest films (i.e., 12.3 μm). This was probably related to a competition of increased amount of reaction sites, hindered analyte penetration, and weakened electrode effects for thicker films. The thickness-optimized WO_3_ films showed excellent NO_2_ selectivity over confounding gases. Furthermore, NO_2_ concentrations were detected down to 3 ppb at 50% RH with a high signal-to-noise ratio (>17), superior to state-of-the-art SMOx-based and commercial NO_2_ sensors. As a result, such morphology-optimized WO_3_ films have a high potential for integration into low power devices for distributed and remote NO_2_ air quality monitoring. In a broader sense, such film morphology optimization could be applied also for other metal-oxide sensors and analytes to improve their sensitivity in environmental monitoring or medical breath analysis [90], where similar challenging sensitivity requirements exist.

## Figures and Tables

**Table 1:** Literature comparison. Selectivity, relative humidity (RH), sensing temperature (T_sensor_), and lower limit of detection (LOD) of state-of-the-art solid-state NO_2_ sensors tested at humid conditions. The LOD represents the lowest concentration measured in the respective study. Responses were linearly interpolated to the same concentrations. Non-conventional abbreviations: RT: room temperature; MeOH: Methanol; EtOH: Ethanol; Ace: Acetone; FA: Formaldehyde.

Material	RH (%) (T_RH_ (°C))	LOD (ppb)	T_sensor_ (°C)	NO_2_ Selectivity									
				H_2_	NH_3_	CH_4_	MeOH	EtOH	Ace	CO	H_2_S	FA	
WO_3_	45 (n/a)	16	150		510					600			[23]
WO_3_	80 (25)	50	200		>10^5^				3000	>10^4^			[21]
WO_3_	40 (25)^c^	40	75		>10^5^			7130			6240		[22]
Fe:WO_3_	45 (n/a)	10	120	185	30				20	185			[12]
In_2_O_3_	25 (n/a)	100	50		10^5^			>10^5^	>10^5^	>10^5^		>10^5^	[24]
SnO_2_/ZnO	10 (20)	50	40				5000	4300	10^4^	>10^4^	4700		[25]
SnO_2_/ZnO	30 (20)	200	RT	>10^5^				>10^4^		>10^5^	6600		[26]
rGO-SnO_2_	25 (n/a)	50	RT		50			100				110	[27]
rGO-Fe_2_O_3_	25 (RT)	100	RT	12	10	16				9			[8]
rGO-CNT-SnO_2_	25 (n/a)	1000	RT		38					77			[9]
**WO_3_**	**50 (23)**	**3**	**125**	**>10^4^**	**>10^5^**	**>10^4^**	**>10^5^**	**>10^5^**	**>10^4^**	**>10^5^**	**835**	**>10^3^**	**This work**

## Supplementary Materials

The following are available online at https://www.mdpi.com/2079-4991/10/6/1170/s1, Figure S1: Sensor chamber, Figure S2: Phase determination by X-ray diffraction, Figure S3: Extended XRD spectrum of the WO_3_ powder, Figure S4: Film thickness determination, Figure S5: Flame-deposited WO_3_ films on Al_2_O_3_, Figure S6: WO_3_ film thickness on Al_2_O_3_ as a function of deposition time, Figure S7: Drift, Figure S8: The effect of higher CO concentrations, Table S1: Optimum WO_3_ temperatures for NO_2_ sensing, Table S2: Porosity evaluation by X-ray diffraction, Table S3: Film thickness effect on selectivity.
