# Supplementary material for: Thickness Optimization of Highly Porous Flame-Aerosol Deposited WO3 Films for NO2 Sensing at ppb
Source: Nanomaterials (Basel). 2020 Jun 16;10(6):1170. doi: 10.3390/nano10061170 (PMC7353271; doi:10.3390/nano10061170)
Supplement: Supplementary file 1 [file nanomaterials-10-01170-s001.pdf]

Supporting Information

Thickness Optimization of Highly Porous Flame-Aerosol Deposited WO_3 Films for NO_2 Sensing at ppb

Sebastian Abegg, David Klein Cerrejon, Andreas T. Güntner and Sotiris E. Pratsinis *

Particle Technology Laboratory, ETH Zurich, Sonneggstrasse 3, CH-8006 Zurich, Switzerland; sabegg@ethz.ch (S.A.); david.klein@pharma.ethz.ch (D.K.C.); andreas.guentner@ptl.mavt.ethz.ch (A.T.G.)
* Correspondence: sotiris.pratsinis@ptl.mavt.ethz.ch (S.E)

Table S1. Optimum WO_3 temperatures for NO_2 sensing. Reported optimum operating temperatures for undoped WO_3 films prepared by wet-phase deposition and sputtering. WO_3 -based sensors are most sensitive to NO_2 at low temperatures, with observed optima typically below 150 ℃, while higher optima were mainly reported for sputtered, thus, dense films. Abbreviations: n/a: not available.

Material	Film fabrication method	Film thickness [µm]	Optimum Temperature [℃]	Ref.
WO_3 hollow microspheres	Coating	n/a	75	[22]
WO_3 yolk-shell spheres	Drop-coating	~12	<100	[21]
WO_3	Printing of a paste	~30	100	[91]
WO_3	Coating	n/a	120	[12]
WO_3	Coating	n/a	125	[46]
WO_3	Spin-coating	n/a	<150	[92]
WO_3	Sputtering	0.085	150	[23]
WO_3 nanorods	Pressing into discs	~500	200	[93]
WO_3	Sputtering	0.360	200	[94]
WO_3	Spin-coating	2.3-3.0	250	[95]
WO_3	Sputtering	0.2 and 0.05	350	[96]

Table S2. Porosity evaluation by X-ray diffraction. Film porosity of flame-deposited WO_3 films on Al_2O_3 substrates evaluated at different XRD peak positions of Al_2O_3. Larger Al_2O_3 substrates (20 x 20 mm^2) were used for the evaluation as the microsensors were too small to be evaluated by XRD.

Deposition time [min]	Porosity evaluated at different Al_2O_3 peak positions (2θ) [%]				
	25.6 °	35.1 °	43.3 °	57.5 °	Avg. ± SD
**1**	97.1	97.0	96.9	96.6	96.9 ± 0.2
**2**	97.7	97.4	97.8	97.7	97.6 ± 0.2
**4**	96.9	96.7	96.7	96.8	96.8 ± 0.1
**8**	97.1	96.9	97.0	96.8	96.9 ± 0.1

**12**	96.9	96.6	96.6	96.5	96.7 ±0.2
**18**	96.9	96.5	96.6	96.5	96.6 ±0.2

Table S3. Film thickness effect on selectivity. NO_2 selectivity comparison over major interferents for different WO_3 film thicknesses. Films were operated at 125 ℃ and 50% RH (at 23 ℃).

**Film thickness [µm]**	**Deposition time [min]**	**NO_2 selectivity ($S_{NO2}/S_{analyte}$) [-]**								
		**H_2**	**NH_3**	**CH_4**	**MeOH**	**EtOH**	**Acetone**	**CO**	**H_2S**	**FA**
0.5	1 min	$>10^5$	$>10^4$	$>10^5$	$>10^5$	$>10^5$	$>10^4$	$>10^5$	415	$>10^5$
3.1	4 min	$>10^4$	$>10^5$	$>10^4$	$>10^5$	$>10^5$	$>10^4$	$>10^5$	835	$>10^3$
12.3	18 min	$>10^3$	$>10^5$	$>10^5$	$>10^5$	795	$>10^5$	$>10^5$	$>10^3$	$>10^3$

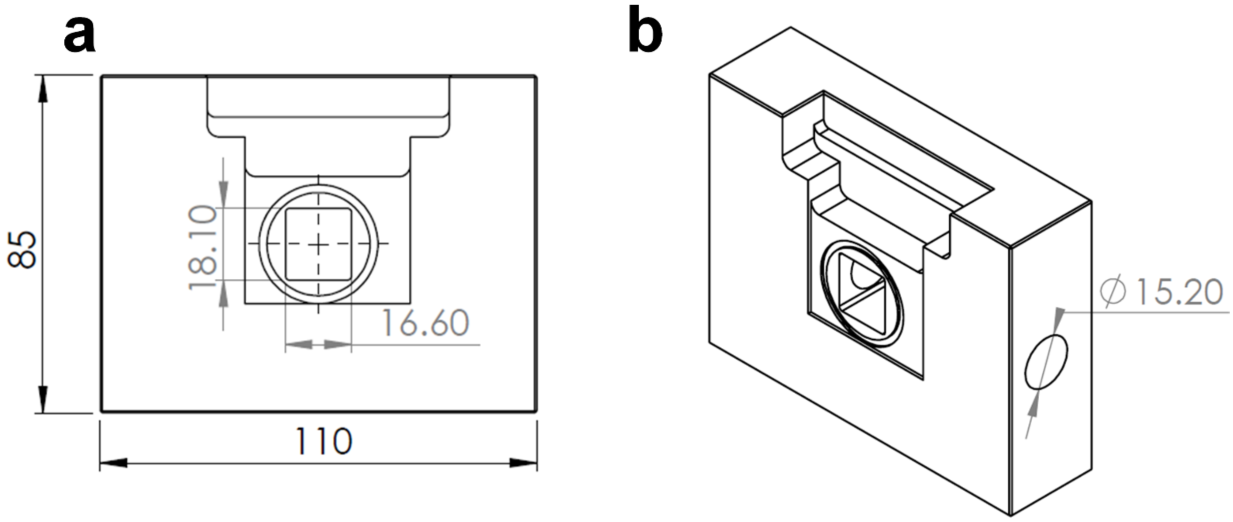

Figure S1. Sensor chamber. (a) Top and (b) 3D view of the sensing chamber (top part) with the sensing cavity (18.1 x 16.6 x 18.0 mm^3) in its center. Recesses accommodate sealings and circuitry for electrical connections to readout and power equipment. The leadless chip carrier containing the microsensors is thereby mounted to a socket soldered onto a printed circuit board [44], that can be attached to the base of the stainless steel sensor chamber (not shown here).

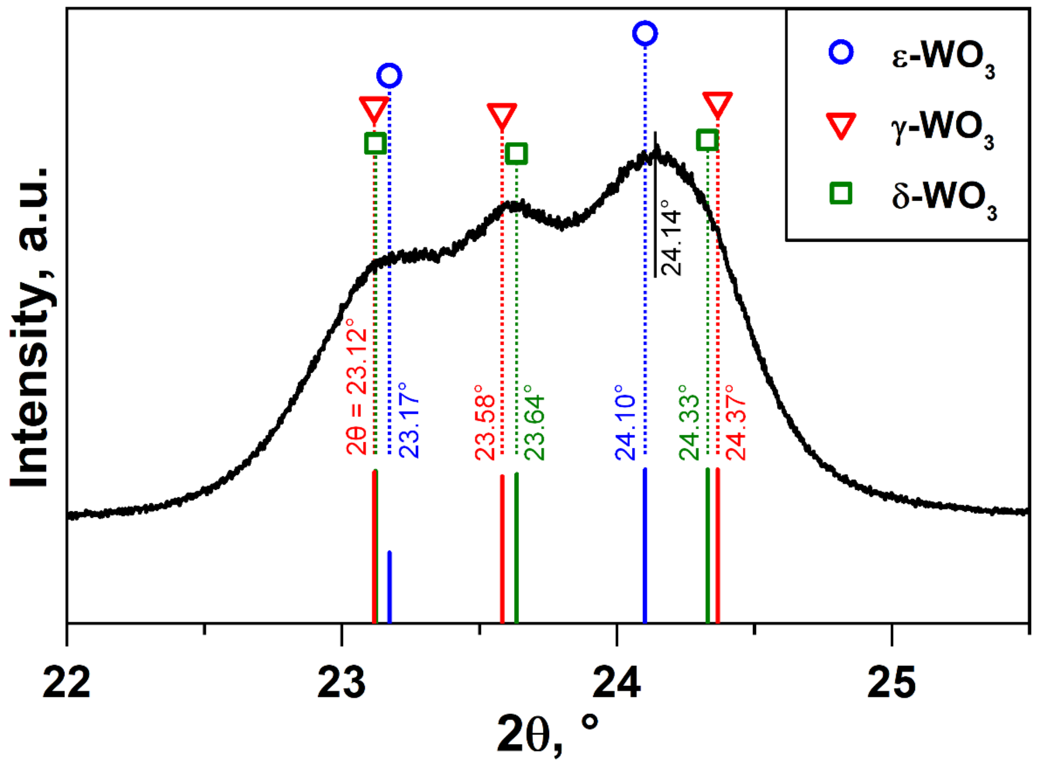

Figure S2. Phase determination by X-ray diffraction. Magnification of the XRD pattern of WO_3 between 2θ = 22 – 25.5°. Reference peaks of ε- (circles, ICSD 84163), γ- (triangles, 80056) and δ-WO_3 (squares, 80053) with corresponding peak positions are indicated.

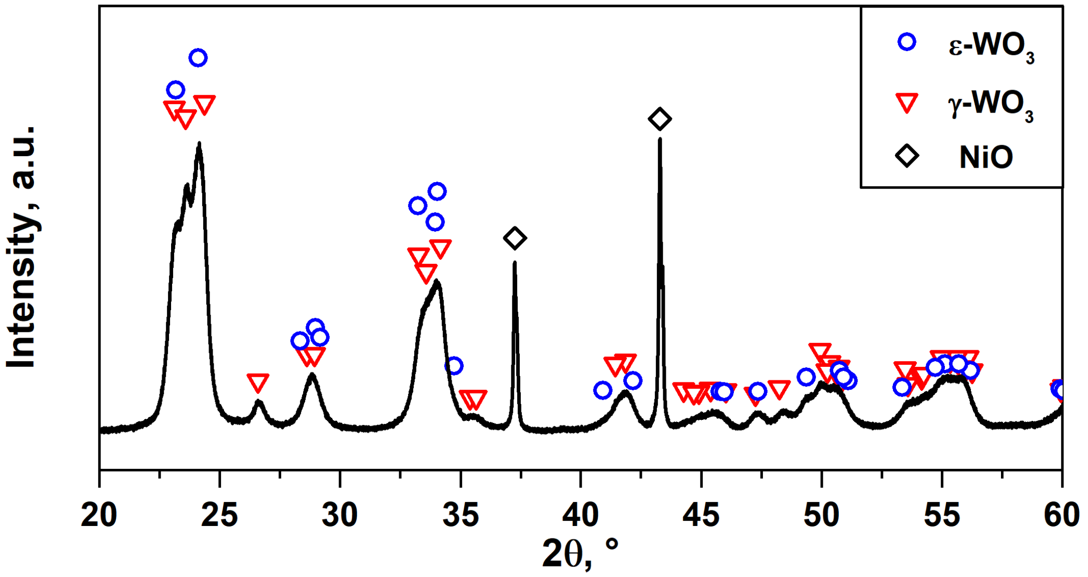

Figure S3. Extended XRD spectrum of the WO_3 powder. Reference peak position for ε- (circles, ICSD 84163), γ-WO_3 (triangles, 80056) and cubic NiO (diamonds, 61324) are indicated. Please note that NiO was added as internal standard for sample displacement correction.

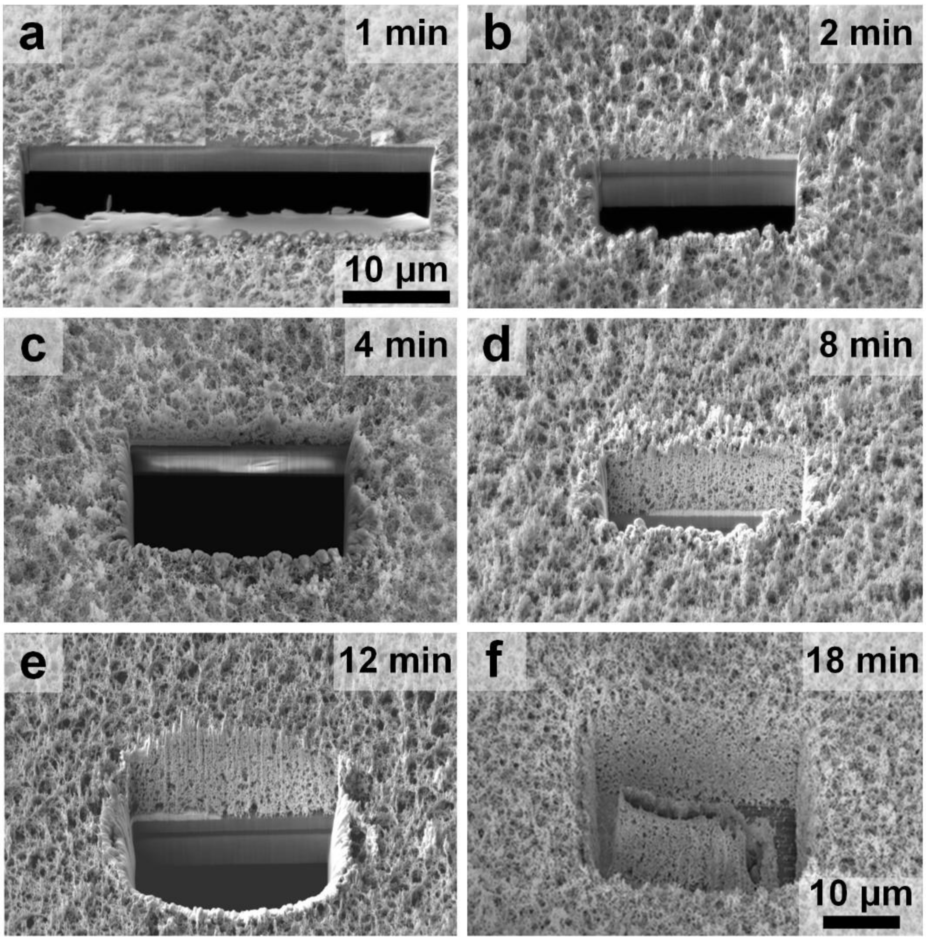

Figure S4. Film thickness determination. FIB-SEM images for (a) 1, (b) 2, (c) 4, (d) 8, (e) 12 and (f) 18 min flame-deposited films. Please note the different scale bar in (f). The more compact structures at the cutting edge are caused by melting during FIB.

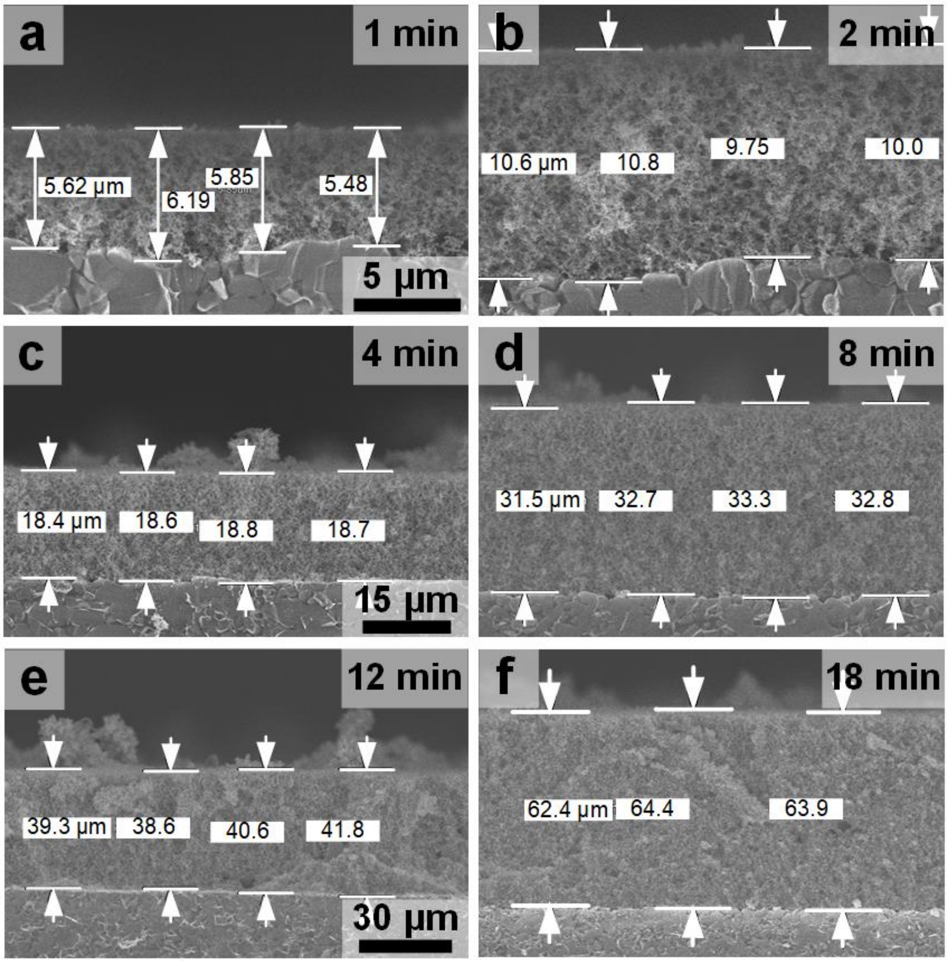

Figure S5. Flame-deposited WO_3 films on Al_2O_3. SEM images for a) 1, b) 2, c) 4, d) 8, e) 12 and f) 18 minutes direct WO_3 particle deposition by FSP on Al_2O_3 substrates along with marks for measuring the average film thickness. Please note the different scale bars that are the same for panels (a,b), (c,d) and (e,f).

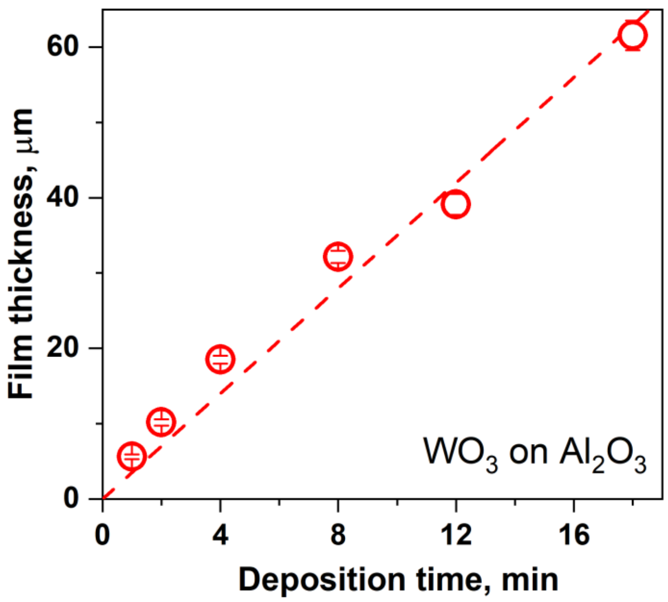

Figure S6. WO_3 film thickness on Al_2O_3 as a function deposition time. Films deposited on Al_2O_3 substrates are thicker (growth rate of 3.50 μm min^{-1}) than on the microsensor ones (Fig. S4) at equal deposition time. This was due to various reasons, like different heat transfer coefficients, absence of the shadowing mask on which significant deposition took place rather than on the microsensor substrate etc. Error bars indicate the variation from 40 different measurements across each substrate.

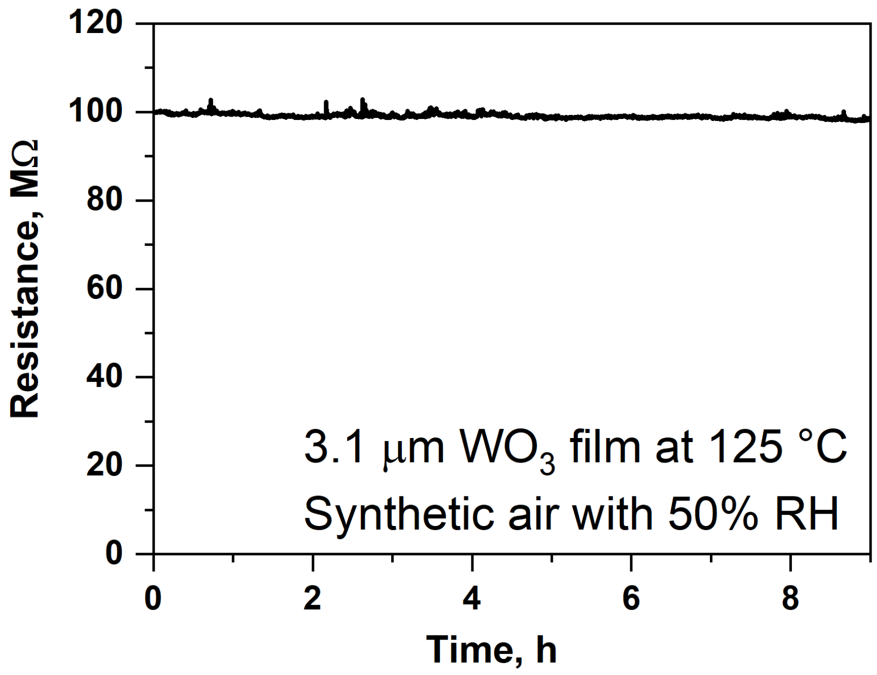

Figure S7. Drift. Baseline stability of a 3.1 μm thick WO_3 film at 125 ℃ in synthetic air with 50% RH.

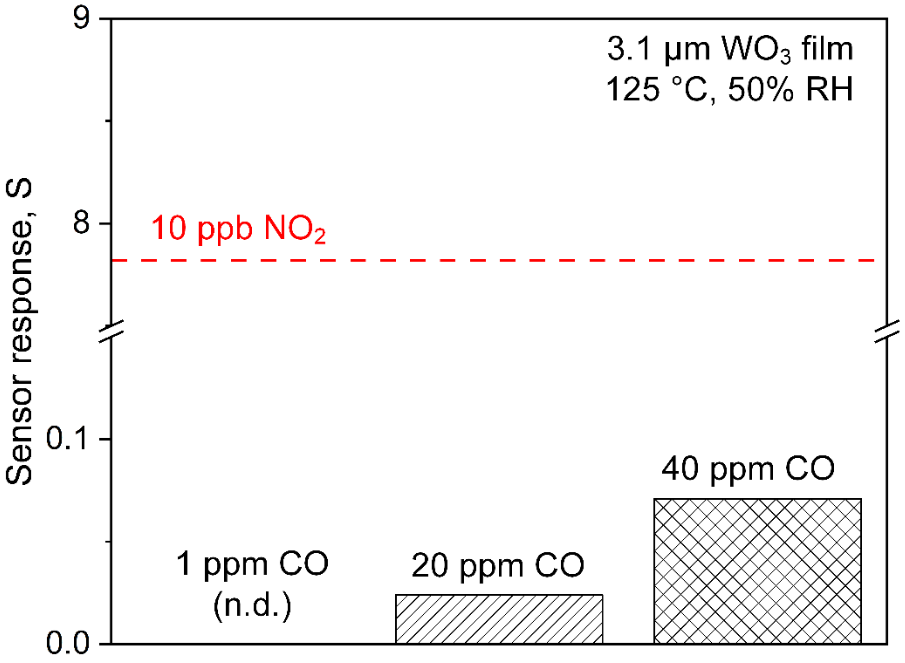

Figure S8. Effect of higher CO concentrations. Sensor responses of the thickness-optimized 3.1 µm WO_3 film to 1, 20 and 40 ppm CO at 50% RH (at 23 °C). For comparison, the response to 10 ppb NO_2 is indicated by a dashed line. Please note the axis break on the ordinate.

Additional references

91. Chung, Y.-K.; Kim, M.-H.; Um, W.-S.; Lee, H.-S.; Song, J.-K.; Choi, S.-C.; Yi, K.-M.; Lee, M.-J.; Chung, K.-W. Gas sensing properties of WO_3 thick film for NO_2 gas dependent on process condition. *Sensor Actuat. B-Chem.* **1999**, *60*, 49-56.
92. Samerjai, T.; Tamaekong, N.; Liewhiran, C.; Wisitsoraat, A.; Phanichphant, S. NO_2 gas sensing of flame-made Pt-loaded WO_3 thick films. *J. Solid State Chem.* **2014**, *214*, 47-52.
93. Bai, S.; Zhang, K.; Luo, R.; Li, D.; Chen, A.; Liu, C.C. Low-temperature hydrothermal synthesis of WO_3 nanorods and their sensing properties for NO_2. *J. Mater. Chem.* **2012**, *22*, 12643-12650.
94. Shen, Y.; Yamazaki, T.; Liu, Z.; Meng, D.; Kikuta, T.; Nakatani, N. Influence of effective surface area on gas sensing properties of WO_3 sputtered thin films. *Thin Solid Films* **2009**, *517*, 2069-2072.
95. Kabcum, S.; Kotchasak, N.; Channei, D.; Tuantranont, A.; Wisitsoraat, A.; Phanichphant, S.; Liewhiran, C. Highly sensitive and selective NO_2 sensor based on Au-impregnated WO_3 nanorods. *Sensor Actuat. B-Chem.* **2017**, *252*, 523-536.
96. Hemberg, A.; Konstantinidis, S.; Viville, P.; Renaux, F.; Dauchot, J.; Llobet, E.; Snyders, R. Effect of the thickness of reactively sputtered WO_3 submicron thin films used for NO_2 detection. *Sensor Actuat. B-Chem.* **2012**, *171*, 18-24.
